# Moxibustion Activates Macrophage Autophagy and Protects Experimental Mice against Bacterial Infection

**DOI:** 10.1155/2014/450623

**Published:** 2014-07-23

**Authors:** Xiaojuan Li, Guanhua Guo, Feng Shen, Lihong Kong, Fengxia Liang, Guojie Sun

**Affiliations:** ^1^Acupuncture and Moxibustion College, Hubei University of Chinese Medicine, Wuhan, Hubei 430061, China; ^2^Hubei Provincial Collaborative Innovation Center of Preventive Treatment by Acupuncture and Moxibustion, Wuhan, Hubei 430061, China; ^3^Shanxi University of Traditional Chinese Medicine, Taiyuan, Shanxi 030024, China

## Abstract

Moxibustion is one of main therapies in traditional Chinese medicine and uses heat stimulation on the body surface from the burning of moxa to release pain or treat diseases. Emerging studies have shown that moxibustion can generate therapeutic effects by activating a series of signaling pathways and neuroendocrine-immune activities. Here we show moxibustion promoted profound macrophage autophagy in experimental Kunming mice, with reduced Akt phosphorylation and activated eIF2*α* phosphorylation. Consequently, moxibustion promoted bacterial clearance by macrophages and protected mice from mortality due to bacterial infection. These results indicate that moxibustion generates a protective response by activating autophagy against bacterial infections.

## 1. Introduction

Autophagy is an evolutionary conserved physiological process by which protein aggregates and damaged organelles inside cells are selectively engulfed and delivered to and degraded in lysosomes [1, 2]. This “self-eating” process is tightly associated with diverse physiological conditions including development and innate defense [3, 4] and a number of pathological conditions and diseases including cancers, neurodegenerative disease, and infectious diseases [5–7]. Emerging studies have shown that modulation of autophagy acts as a potential therapeutic strategy for treatment of disease [8, 9], even though autophagy is a double-edged sword in the progression of cancer and certain metabolic diseases [10–12]. Autophagy may restrict bacterial replication in the cytoplasm by clearance or be hijacked to support bacterial replication and survival [13, 14], a unique evasion technique of bacteria, which uses a variety of machinery to evade host defense systems to facilitate survival inside cells [15]. Autophagy primarily acts as an innate defense against bacterial infection [16], and enhanced autophagy provides a protective response against bacterial and virus infection [17, 18]. However, it is difficult to modulate autophagy in unique lesions* in vivo*, which limits the application of autophagy as a therapeutic strategy.

Moxibustion is a physical therapy in traditional Chinese medicine that uses heat from the burning of minced herb moxa to stimulate acupuncture points for pain relief or disease treatment [19–21]. Moxibustion can affect neuroendocrine-immune functions and activate self-healing [22, 23]. Although the substitution of moxibustion by thermal physical therapy remains equivocal [23, 24], they both share the similar mechanism in self-healing and pain reduction [25, 26]. Emerging studies have shown that moxibustion alters the profile of gene expression, including cytokines [27–30] and signaling pathways [31–33], indicating it might be beneficial to patients with autoimmune and inflammatory diseases.

Previously, a protective response of moxibustion against HSV-1 infection was demonstrated in experimental mice [34]. However, the mechanisms of how moxibustion might be useful against infectious diseases remain to be fully defined. The present study shows that moxibustion activates autophagy of intraperitoneal macrophages in experimental Kunming mice, following the inactivation of Akt and the induction of eIF2*α* phosphorylation. Consequently, it contributes to a protective response against bacterial infection, indicating moxibustion activates innate defenses to prevent infectious disease.

## 2. Methods and Materials

### 2.1. Mice and Ethics Statement

Specific-pathogen-free male Kunming mice (6–8 weeks of age, 18–22 g weight) were maintained and bred according to the guidelines approved by the Laboratory Animal Center, Sun Yat-sen University.

### 2.2. Mild Moxibustion on Lethal Infections of* Staphylococcus aureus*

Forty mice were randomly divided into four groups, A, control group; B, model group; C, 15 min moxibustion group; and D, 30 min moxibustion group, with 10 mice per group. Except for the control group, the remaining 30 mice were primed with intraperitoneal injection of 6% starch broth for 2 days, followed by intraperitoneal injection of 10 LD_50_ *Staphylococcus aureus* as an acute infection model. One hour after injection with bacteria, mice in groups C and D were restrained and administered *ϕ*4 mm stick moxibustion for 15 min and 30 min at Guanyuan acupuncture point (CV 4) at the front of the small intestine. In the control group and model group, *ϕ*4 mm sticks of unlighted moxa herbs were used as the control for moxibustion. The death of mice was monitored continuously for 48 h after injection to prepare a survival curve. Peritoneal fluids were collected immediately upon death or sacrifice at 48 h after moxibustion and subjected to bacterial colony formation inLuria-Bertani (LB) agar plates. The numbers of colonies were used to evaluate bacterial clearance.

### 2.3. Mild Moxibustion on Macrophage Autophagy

Forty mice were randomly divided into four groups: A, control group; B, model group; C, 15 min moxibustion group; and D, 30 min moxibustion group. In B, C, and D groups, mice received intraperitoneal injection of 6% starch broth as a macrophage inducer, and two days later mice in C and D groups were administered moxibustion at CV4 as described above and sacrificed at 1 h after moxibustion. Peritoneal fluid cells were collected and subjected to autophagy assays. To detect the dynamic Akt and eIF2*α* phosphorylation under longer moxibustion, an additional group with 60 min moxibustion was performed following the procedure above.

### 2.4. Assays of Autophagy and Autophagic Pathways

A small number of fresh macrophages were fixed with 3% formaldehyde in phosphate buffer (PBS) for 10 min and permeabilized with 0.5% Triton X-100 in PBS for 10 min. After washing with PBS twice, the cells were blocked with 1% bovine serum albumin in PBS with 0.1% Triton X-100 for 30 min and then incubated with rabbit polyclonal anti-LC3 antibody (1 : 200, MBL International, MA, USA) for 1 h. After three washes with PBS with 0.1% Triton X-100, the cells were incubated with FITC-conjugated anti-rabbit IgG secondary antibody (1 : 500, Life Technologies) for 0.5 h, counterstained with DAPI (4′,6-diamidino-2-phenylindole, Sigma, St. Louis, MO), and mounted in antifade agent (Invitrogen, Carlsbad, CA). Finally, LC3 staining was visualized by confocal fluorescence microscopy. The remaining macrophages were lysed and total protein extracts were subjected to western blots with anti-LC3 antibody, phospho-eIF2*α* (Ser51) rabbit monoclonal antibody (mAb), phospho-Akt (Ser473) rabbit mAb, and anti-Akt and eIF2*α* rabbit antibodies (1 : 1000, Cell Signaling Technology, MA, USA). Images were visualized by Licor Odyssey or Pierce ECL system.

### 2.5. Statistical Analysis

The survival rates were analyzed by a log-rank test; and the statistical analysis was performed with one-way analyses of variance (ANOVA).

## 3. Results

### 3.1. Moxibustion Promotes Autophagy in Macrophages

**Figure 1:**
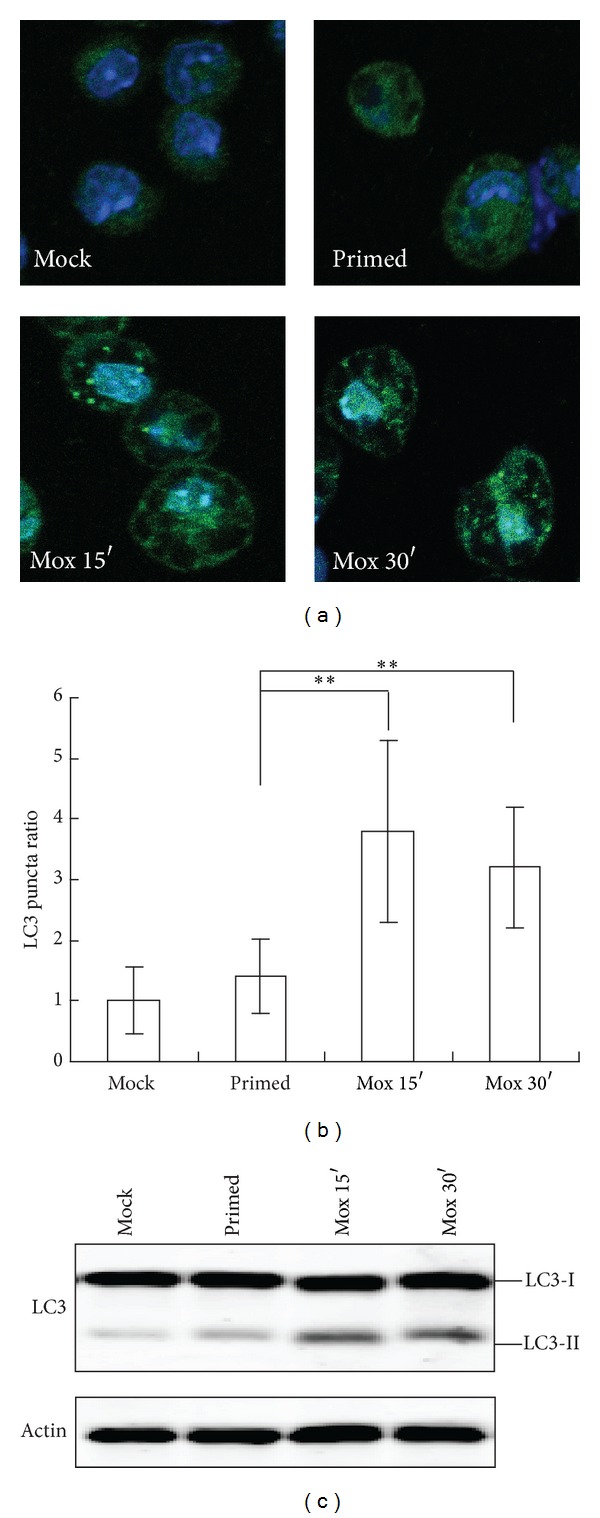
Autophagy is accelerated by moxibustion. Macrophages were collected from mice with or without moxibustion as indicated, fixed, and stained with anti-LC3 antibody, and LC3 staining was visualized by confocal fluorescence microscopy. Representative images (a) and the means of LC3 puncta/cell counted from 20 individual cells (b) are shown. **P < 0.01. Macrophages were lysed and equal mixtures of whole cell extracts were detected by western blots with anti-LC3 and anti-actin antibodies (c).

To assess whether moxibustion affected autophagy, moxibustion with a small size moxa stick was applied to the mouse abdomen Guanyuan point (CV4) for 15 and 30 minutes. Macrophages were then isolated from the peritoneal fluid after mice were sacrificed and subjected to autophagy assay. When macrophages were primed by starch and albumen, autophagic LC3-postive vacuoles were not observed in macrophages, similar to that seen in control mice, whereas LC3-positve puncta were greatly increased after 15 min or 30 min moxibustion (Figures 1(a) and 1(b)). Furthermore, when LC3 was detected in cell extracts, the ratio of LC3-II was elevated following moxibustion compared with mock and primed conditions (Figure 1(c)). The levels of LC3-II protein and LC3 puncta were similar under both moxibustion conditions. These results suggest that moxibustion induces the macrophage autophagy in mice.

### 3.2. Moxibustion Induces eIF2*α* Phosphorylation and Suppresses Akt Phosphorylation

**Figure 2:**
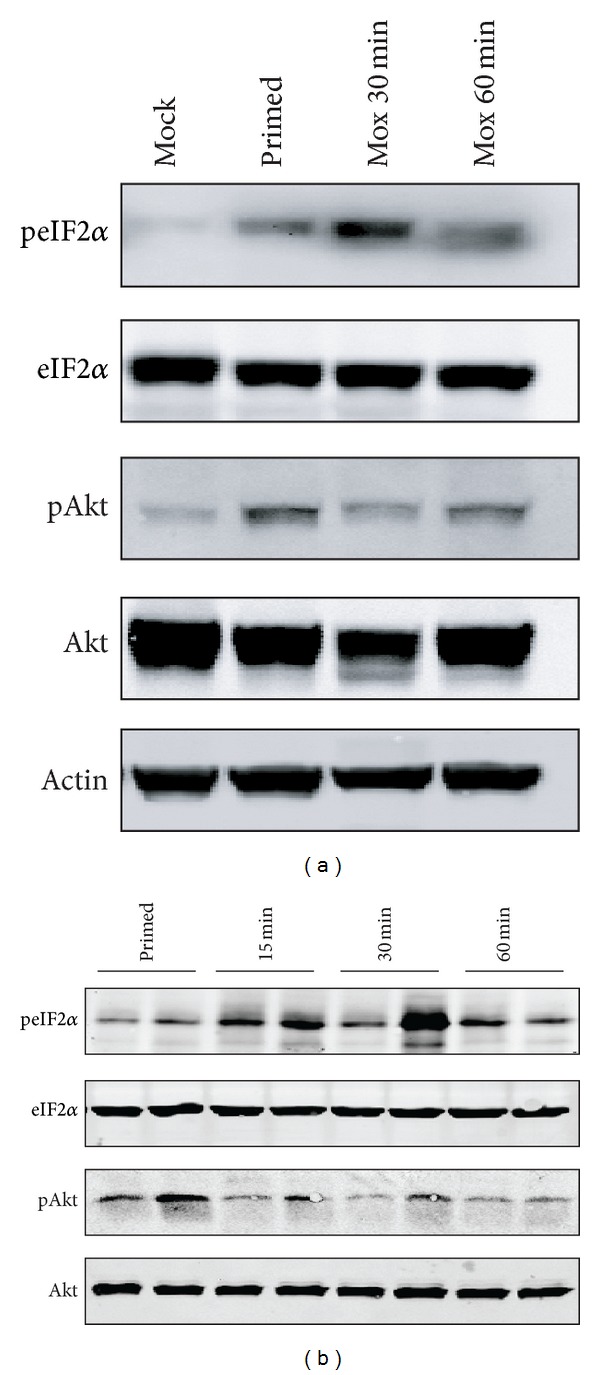
Autophagic Akt and eIF2*α* pathways are activated by moxibustion. (a) Four mice per group were untreated or treated as indicated and equal whole cell extracts of macrophages from mice were mixed and signaling pathways detected as indicated. (b) Two random macrophage samples from six individual mice per group are shown.

We attempted to address upstream signaling pathways by which moxibustion activates autophagy. Akt inhibits endoplasmic reticulum (ER) stress by phosphorylating eIF2*α*-kinase PERK [35] and the PI3K/Akt/mTOR pathway acts as a well-characterized negative regulator of autophagy [36]. Because moxibustion delivers heat stimulation to the operative site, we hypothesized it induced a stress response, and then Akt activity and ER stress were examined after moxibustion. To diminish the individual differences in mice, we investigated ER stress in mixed macrophages (Figure 2(a)) and random macrophages (Figure 2(b)). As shown in Figures 2(a) and 2(b) in top panel, phosphorylation of eIF2*α* in macrophages was significantly elevated at 30 min and then slightly fell back at 1 h during moxibustion, indicating a rapid ER stress response was activated. Furthermore, the phosphorylation of Akt was decreased dramatically at both 30 min and 1 h, suggesting moxibustion suppressed Akt activation (Figures 2(a) and 2(b) in bottom panel). Thus, we concluded that moxibustion induced autophagy in macrophages by suppressing the Akt pathway and activating ER stress.

### 3.3. Moxibustion Protects Experimental Mice from Bacterial Infection

**Figure 3:**
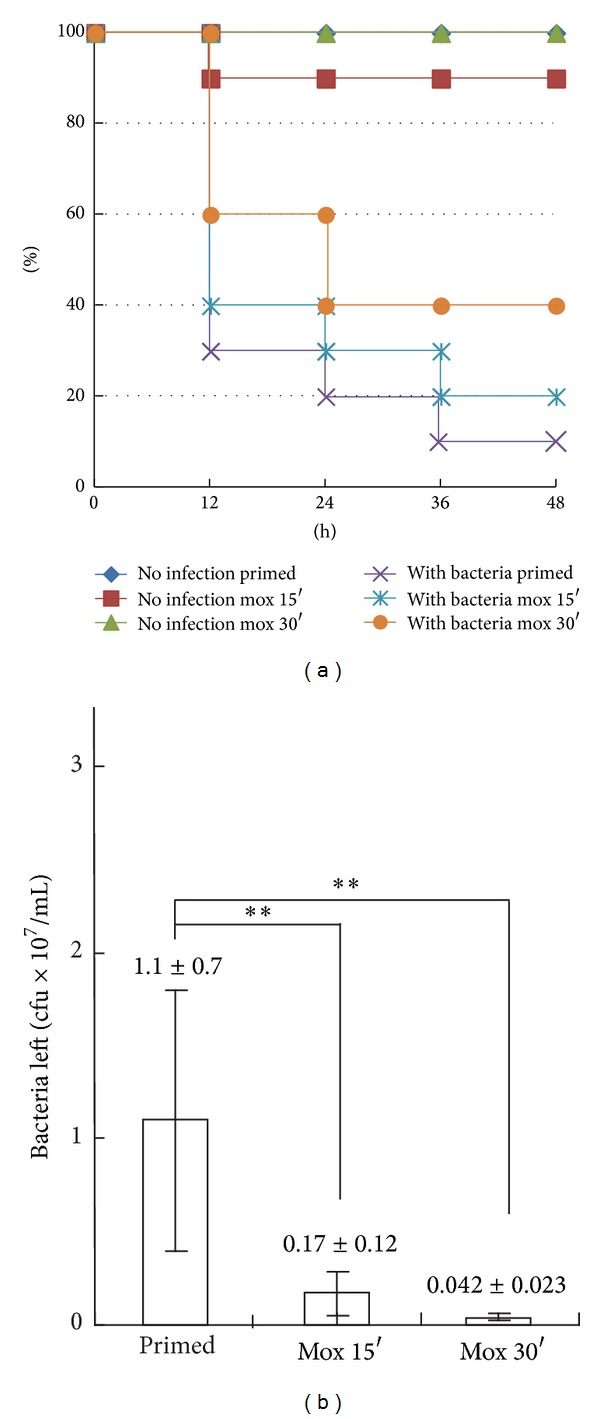
Bacterial infection is alleviated by moxibustion. (a) Mice were injected intraperitoneally with 10 LD_50_ *Staphylococcus aureus* as an acute bacterial model. At 1 h later, mice were untreated or administered moxibustion as indicated. The parallel tests without bacterial infection were performed following the same procedures. Survival was monitored for 48 h after moxibustion (*n* = 10 mice/group) and the log-rank test was performed for the survival curve. *P* < 0.05, primed group versus 30 min moxibustion group. (b) Once mice died or were sacrificed at 48 h after moxibustion, peritoneal fluids were collected immediately and plated on LB agar to measure the activity of bacterial colony formation. **P < 0.01.

Because elevated autophagy promotes bacterial clearance of macrophages, we hypothesized moxibustion would protect mice against bacterial infection with* Staphylococcus aureus. *Bacterial challenge caused 90% mortality in primed mice at 48 h, during which the bacteria did not escape from killing by macrophages postinfection [37, 38]. The group receiving 15 min moxibustion showed little difference in survival compared with the control group, whereas the 30 min moxibustion group was significantly protected from death and acute infection (Figure 3(a)). This suggested the 30 min moxibustion induced a protective effect against bacterial infection. Furthermore, remaining bacteria were detected after moxibustion. As shown in Figure 3(b), the bacterial loads in the peritoneal fluid were dramatically reduced when mice were administered moxibustion for 15 min or 30 min, although the latter was more effective. In conclusion, moxibustion provides a protective response and enhanced clearance of bacterial infection in macrophages.

## 4. Discussion

In the present study, we revealed that macrophage autophagy in the peritoneal fluid was elevated when moxibustion was applied to experimental mice. Moxibustion provided a protective response against bacterial infection. This effect probably contributes to the physiological stress response with Akt inhibition and activation of ER stress.

Moxibustion is one physical therapy in traditional medicine that utilizes heat stimulation to cure disease or quell pain. It might induce a series of signal pathways and stress through heat shock proteins [22, 39], including the Akt pathway and ER stress, as shown in the present study. Although the effect of moxibustion in the whole body requires more evidence, moxibustion is a suitable technique for local heat therapy to stimulate self-healing, recovery, and innate defense mechanisms. It is important to prevent severe heat stimuli by moxibustion that can cause burn injury following inflammation. However, heat therapy is beneficial when the heat stimulation is artificially controlled to a unique site without injury [40, 41].

Even though the effects of moxibustion have not yet been systemically evaluated, emerging studies show moxibustion can alter gene expression and signal transduction. The long-distance effect of moxibustion may be due to neural reflexes [25], whereas local effects are mediated through cytokines and signal transduction. The present study and previous studies have revealed moxibustion activates a unique cytokine profile and signal transduction pathways [22, 27–34, 42, 43], as well as elevating macrophage autophagy (Figure 1) and natural killer cell activity [34]. These results suggest moxibustion generates a protective response against viral and bacterial infection, although it is not clear whether it functions locally or systemically.

Although the studies have shown moxibustion contributes to the systemic and comprehensive immune, inflammatory, and analgesic activities, our finding represents a novel protective mechanism that the mild moxibustion promotes the autophagy and bactericidal function of macrophage. This effect is probably contributed to the inhibition of Akt phosphorylation and the activation of eIF2*α* phosphorylation, two key signal pathways in a variety of stresses. However, the upstream signaling of moxibustion remains elusive and the further systematic analysis regarding metabolism and endocrine under moxibustion will greatly help to understand the mechanism. Alternatively, it is urgent to improve the clinical efficacy of moxibustion as well as to definete the indications and usage.

## 5. Conclusion

Our data provide evidence that moxibustion augments autophagy in macrophages and innate defense against bacterial infection. Based on our present study, mild moxibustion is effective at preventing and treating infectious disease in mice, acting as a simple and convenient activator of autophagy in physical therapy.
